# Applications of plant‐derived extracellular vesicles in medicine

**DOI:** 10.1002/mco2.741

**Published:** 2024-09-20

**Authors:** Yawen Zhu, Junqi Zhao, Haoran Ding, Mengdi Qiu, Lingling Xue, Dongxue Ge, Gaolin Wen, Haozhen Ren, Peng Li, Jinglin Wang

**Affiliations:** ^1^ Division of Hepatobiliary and Transplantation Surgery Department of General Surgery Nanjing Drum Tower Hospital Clinical College of Nanjing University of Chinese Medicine Nanjing China; ^2^ Department of Cardiology The First Affiliated Hospital of Nanjing Medical University Nanjing Jiangsu China

**Keywords:** biomedical application, clinical treatment, delivery strategy, extracellular vesicles, plant

## Abstract

Plant‐derived extracellular vesicles (EVs) are promising therapeutic agents owing to their natural abundance, accessibility, and unique biological properties. This review provides a comprehensive exploration of the therapeutic potential of plant‐derived EVs and emphasizes their anti‐inflammatory, antimicrobial, and tumor‐inhibitory effects. Here, we discussed the advancements in isolation and purification techniques, such as ultracentrifugation and size‐exclusion chromatography, which are critical for maintaining the functional integrity of these nanovesicles. Next, we investigated the diverse administration routes of EVs and carefully weighed their respective advantages and challenges related to bioavailability and patient compliance. Moreover, we elucidated the multifaceted mechanisms of action of plant‐derived EVs, including their roles in anti‐inflammation, antioxidation, antitumor activity, and modulation of gut microbiota. We also discussed the impact of EVs on specific diseases such as cancer and inflammatory bowel disease, highlighting the importance of addressing current challenges related to production scalability, regulatory compliance, and immunogenicity. Finally, we proposed future research directions for optimizing EV extraction and developing targeted delivery systems. Through these efforts, we envision the seamless integration of plant‐derived EVs into mainstream medicine, offering safe and potent therapeutic alternatives across various medical disciplines.

## Graphical abstract

This review provides a comprehensive review of the state‐of‐the‐art treatment for diseases using exosomes derived from plant. By understanding the composition of exosomes, methods of administration, and their clinical applications, the aim is to explore their therapeutic potential and establish a basis for improving health.

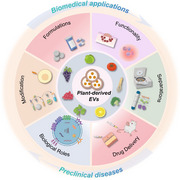

## 1 INTRODUCTION

Plants, as natural sources of medicine, have significant potential for disease treatment owing to their abundant resources and easy accessibility.^1^, ^2^ Various plants possess anti‐inflammatory, antimicrobial, and tumor‐inhibiting effects, strongly supporting the research and development of plant‐based medicines.^3^, ^4^ For example, the active ingredients in turmeric and dandelion can inhibit inflammatory responses and alleviate related disease symptoms.^5^, ^6^ Artemisinin is used to treat tuberculosis infection because its effective components can eradicate this deadly pathogen.^7^ Additionally, some plants have applications in cancer treatment, for example, certain compounds in *Tripterygium wilfordii* with tumor growth inhibition properties.^8^ Despite this progress, traditional methods for preparing plant‐based medicines retain the complexity of their natural components. This complexity can lead to toxicity and side effects, even when the therapeutic effects are produced.^9^ Furthermore, the unclear treatment mechanisms of plant medicines make it challenging to understand their actions and associations with diseases. Therefore, novel strategies are required to extract and purify effective components from plant‐based medicines to elucidate their treatment mechanisms and expand the prospects of their application in disease treatment.^10^, ^11^

The emerging concept of exosomes has sparked interest in their application in plant‐based medicine. Exosomes, widely distributed extracellular vesicles (EVs) in organisms, serve as natural conveyors for a variety of biomolecules. Their stable lipid membrane structure enables the secure delivery of bioactive molecules to damaged cells.^12^, ^13^ Owing to their small size and biological composition, EVs can efficiently traverse cell membranes, thereby enhancing drug absorption and distribution. Additionally, exosome extraction and refinement are relatively feasible, ensuring compliance with pharmaceutical production standards.^14^ Furthermore, bioactive molecules encapsulated within EVs are typically more targeted and precise, thereby facilitating enhanced precision in disease treatment. Although significant research has focused on EVs derived from mammalian cells, plant‐derived EVs have also garnered attention because of their distinct characteristics. Plant‐derived EVs can evade the detection of immune function and therefore have a longer duration of action in the body.^15^, ^16^ Furthermore, plants cannot harbor zoonotic or human pathogens like mammals, making plant‐derived EVs nonimmunogenic and safe for use.^17^, ^18^ Therefore, understanding the mechanisms of action, distribution patterns, and therapeutic potential of plant‐derived EVs is vital for fully harnessing the capabilities of these natural nanovesicles in medical applications.

**FIGURE 1:**
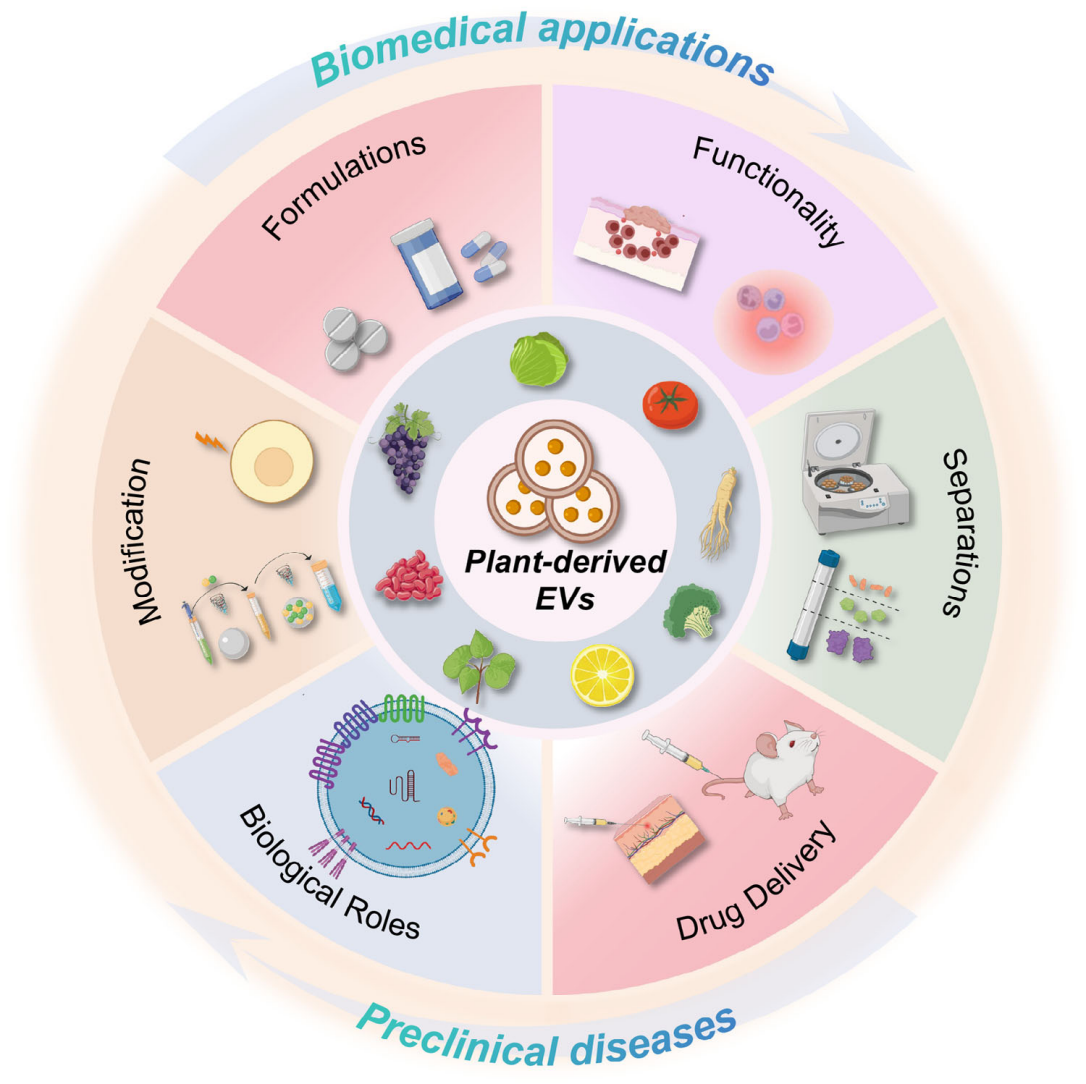
Schematic representation of extracellular vesicles derived from plant and their applications for disease treatment (created by BioRender.com and MedPeer).

Here, we provide a comprehensive summary of the research progress in plant‐derived EVs and offer insights into their applications in medicine (Figure 1). We detail their isolation and purification methods, discussing various techniques such as ultracentrifugation, polyethylene glycol (PEG)‐based precipitation, and size‐exclusion chromatography.^19^, ^20^ Furthermore, we introduce the sources of different types of EVs, highlighting both natural and modified plant‐derived vesicles. These methodologies ensure the purity, functionality, and stability of the isolated EVs, which are crucial for their efficacy in therapeutic applications. Further, we summarize relevant literature on the various routes of EV administration, including intravenous, oral, in situ, intraperitoneal, and subcutaneous injections. Each of these routes was analyzed for its advantages and disadvantages in terms of absorption, distribution, bioavailability, and duration of action. For instance, intravenous administration allows for rapid systemic distribution but may induce immune reactions, whereas oral administration is noninvasive and patient friendly; however, it faces challenges related to stability in the gastrointestinal tract.^21^, ^22^ Moreover, we extensively discuss the mechanisms of plant‐derived EVs in biomedical applications and summarize four main mechanisms: anti‐inflammatory, antioxidant, antitumor, and maintenance of gut microbiota. We then detail the mechanisms by which EVs from specific plant sources affect diseases such as cancer, ischemia–reperfusion injury (IRI), wounds, and colitis. Finally, we discuss the challenges of plant‐derived EV in disease treatment, including the scalability of production, regulatory hurdles, and ensuring consistent quality and efficacy. We also examined issues related to stability, delivery, and potential immunogenicity. To address these challenges, we propose future research directions aimed at optimizing the extraction and purification processes, developing targeted delivery systems, and conducting extensive preclinical and clinical trials. Our vision for the future of plant‐derived EVs includes integration into mainstream medical practices and leveraging their natural properties for safe and effective disease treatments. By overcoming the current obstacles and continuing to elucidate their mechanisms of action, plant‐derived EVs hold great promise for advancing therapeutic strategies in various medical fields.^23^, ^24^

## 2 ISOLATION AND CHARACTERIZATION OF PLANT‐DERIVED EVs

To obtain EVs, it is essential to isolate and purify plant components. Currently, several methods are available for isolating and purifying EVs from natural medicinal plants, including ultracentrifugation, PEG‐based precipitation, and size‐exclusion chromatography. Ultracentrifugation has also been used in many studies.^25^ However, there is currently no widely standardized experimental procedure for a specific isolation or purification method. Therefore, it is crucial to summarize and thoroughly elucidate the potential of different EVs acquisition methods in medical applications.

### 2.1 Isolation plant‐derived EVs

Ultracentrifugation is a popular and straightforward strategy for separating EVs from other proteins using different centrifugal forces. This method involves removing cellular debris by centrifugation at low speed (300×*g*), followed by ultra‐high‐speed centrifugation at 100,000×*g* to separate the supernatant and retain the precipitate.^26^, ^27^ Although ultracentrifugation is widely accepted as the gold standard for experimental EVs isolation, it has some limitations. For instance, ultracentrifuges are bulky and expensive, centrifugation is time consuming, and there is a risk of contamination with aggregated proteins and ribonucleoprotein particles. Moreover, this method may not be suitable for microvolume samples because of their high sample volume required.^28^ Additionally, when dealing with plant samples, such as Pueraria lobata and yam, which are more viscous or contain more impurity particles, the separation efficiency of ultracentrifugation may be relatively low.^29^, ^30^

PEG‐based precipitation is a commonly used ultracentrifugation method. The principle behind this method is that PEG binds to and coprecipitates with hydrophobic proteins and lipid molecules, rendering exosomes less soluble in the PEG solution and causing their precipitation, thus facilitating EVs isolation.^31^ Typically, PEG is added to a liquid sample containing EVs and then centrifuged at low speed. The kits based on this method are readily available. PEG‐based precipitation is known for its ease of use and time‐saving nature.^32^, ^33^ Despite its convenience, there are some technical challenges that are difficult to overcome, such as low purity, high presence of impure proteins, and difficulty in removing biopolymers. Jokhio et al.^34^ successfully isolated EVs from *Arabidopsis thaliana* leaves using PEG precipitation. They employed dynamic light scattering (DLS) to determine the average particle size, which was consistent with the nanoparticle tracking analysis (NTA). In summary, PEG precipitation is a convenient, yet technically challenging method for EV isolation.^35^

Size‐exclusion chromatography relies on the size of the EVs used for separation. In this method, plant samples are passed through porous polymer microspheres. Larger particles are eluted directly through the spaces between the microspheres, whereas smaller particles diffuse through them, elute more slowly, and eventually exit the column. Recently, this method has been used for exosome isolation. EVs obtained by size‐exclusion chromatography from complex biological cultures exhibit high purity with minimal impurity proteins.^36^, ^37^ However, factors such as media type, pore size, and vesicle–media interaction must be considered when passing the sample through the column, as fluid flow may cause crushing and distortion, potentially affecting the integrity of exosomes. Kim et al.^38^ introduced large‐scale silicon nitride nanosieves, scaled up to 4‐in. wafers, to enhance the nanoparticle permeability. This approach resulted in up to 90% recovery of intact exosomes with a purity ratio of 3 × 10^7^ particles/µg protein, which is 4.6‐fold higher compared with ultracentrifugation.^38^

### 2.2 Characterization of plant‐derived EVs

The particle size and potential were characterized using DLS and NTA.^39^, ^40^ Different EV types have varying particle sizes and negative zeta potentials. DLS determines the average particle size by analyzing light intensity fluctuations caused by Brownian motion.^41^ It requires a small sample size for accurate measurements in monodisperse systems but offers limited resolution. In contrast, NTA can visualize and quantify individual EVs, providing concentration data and accurately representing the vesicle status. NTA is ideal for particle size and concentration assessments in complex systems but requires the sample to be within a specific concentration range for measurement. The NTA fluorescence mode enables the tracking of specific EV phenotypes, aiding in background interference elimination.^42^ Ultimately, these techniques perform an essential function in effectively understanding and analyzing EV properties.

Electron microscopy techniques, including scanning electron microscopy (SEM), transmission electron microscopy (TEM), and cryoelectron microscopy (cryo‐EM), can be used to analyze the morphology of EVs.^43^, ^44^ SEM was primarily employed to observe the surface structure of the samples, whereas TEM was used to visualize the internal structure and particle morphology. Notably, the dehydration process during sample preparation for TEM may lead to sample shrinkage and alterations in the actual morphology. Nonetheless, TEM remains a widely used technique for achieving nanoscale resolution. In contrast, cryo‐EM is capable of studying EV morphology at low temperatures, thereby circumventing the issues associated with morphological changes caused by dehydration.^45^ In conclusion, these techniques revealed morphological similarities between plant‐ and mammalian‐derived EVs, both exhibiting a spherical shape with surface depressions that may be linked to the preparation process.

Surface marker proteins are pivotal in identifying EVs. CD9, CD63, CD81, and TSG101 are among the most common protein markers found in EVs and that the protein composition of EVs can vary among different plant species.^46^, ^47^ The specific protein composition of EVs is determined by factors such as growth conditions and surrounding matrix. Additionally, proteomic studies have confirmed the significance of EV protein composition in medicinal plants. For example, Hao et al.^48^, ^49^ identified various protein families in plant‐derived EVs, including proteins found in mammals, such as those involved in protein hydrolysis and synthesis, as well as membrane‐bound proteins, lattice proteins, and small GTPases. Furthermore, plant‐specific proteins, such as chloroplast and cell wall‐associated proteins, have been detected. Despite the diverse protein profiles identified in plant‐derived EVs, investigations on specific protein markers are ongoing.

## 3 TYPES OF PLANT‐DERIVED EVS

Currently, there are two main types of plant‐derived EVs, natural and modified. Natural plant‐derived EVs include various herbs sourced from the roots, leaves, vegetables, fruits, and barks of plants.^50^, ^51^ Plant‐derived EVs possess inherent anti‐inflammatory and antioxidant properties. Herbal remedies have a rich history in Asia, particularly China, where traditional methods and techniques for disease treatment have been refined over the past millennia.^52^, ^53^ However, the intricate nature of herbal ingredients, complex processing procedures, inconsistent dosages, and low patient adherence hinder the realization of the therapeutic potential.^54^ Recently, researchers have enhanced the therapeutic efficacy of plant‐derived EVs by modifying them for drug delivery purposes.^55^, ^56^

### 3.1 Natural plant‐derived EVs

Unlike chemically synthesized drugs, natural medicines often exhibit multiple targeted effects and can simultaneously regulate multiple biological pathways for the treatment and prevention of diseases. This multitargeting capability helps minimize side effects and enhances therapeutic efficacy. Natural medicines contain multiple active ingredients that can synergistically reduce toxicity and enhance efficacy.^57^, ^58^ Natural medicines also offer personalized treatment options tailored to individual patient needs, thereby optimizing therapeutic outcomes and safety.^59^, ^60^ Specifically, Chinese medicine contains a diverse array of active ingredients, such as saponins, peptides, enzymes, phenols, and quinones, which possess various biological activities, including promoting wound healing, enhancing cellular activity, and providing anti‐inflammatory and antioxidant effects.^61^ These active ingredients serve as the foundation for the therapeutic effects of Chinese medicine.^62^, ^63^

Natural medicinal plants play an important role in drug purification, and EVs extracted from plants are widely recognized as natural drug carriers because of their wide distribution, nonimmunogenicity, and targeted delivery properties. The use of EVs to transport the active ingredients of traditional Chinese medicines can target drug delivery to specific sites and improve bioavailability and efficacy.^64^, ^65^ Plant‐derived EVs have a lipid bilayer similar to that of liposomes commonly used in skin care products, which is an effective transdermal system that penetrates deep into the stratum corneum. The mediated delivery of active ingredients effectively utilizes the unique multitarget, multipathway therapeutic approach of traditional Chinese medicine to achieve the comprehensive treatment of diseases.^66^, ^67^

#### 3.1.1 Pueraria lobata

**FIGURE 2:**
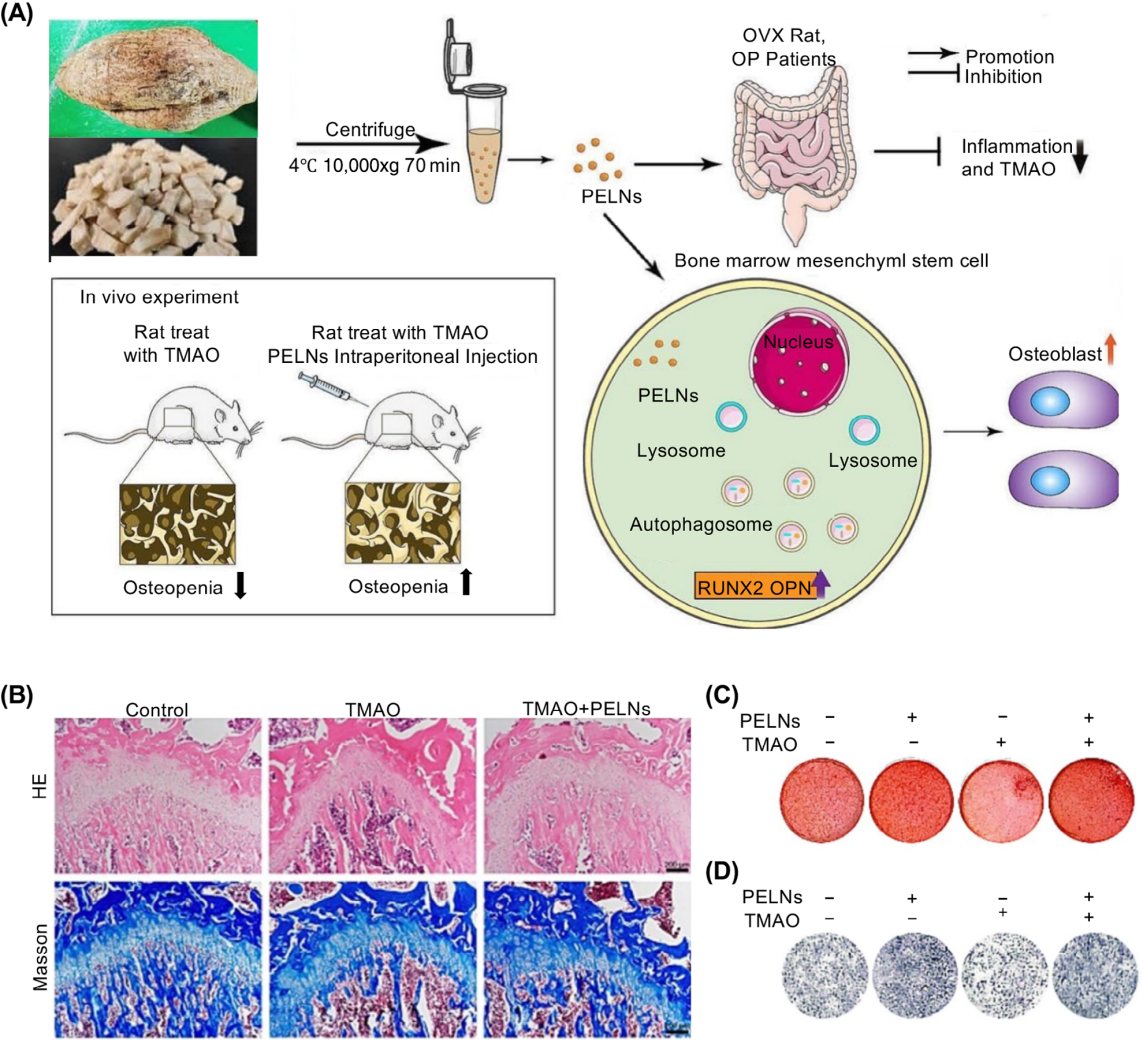
Schematic illustration of *Portulaca oleracea* L.‐derived exosome‐like nanoparticles (PELNs) alleviate osteoporosis by enhancing autophagy. (A) Isolation of exosomes from *Pueraria lobata* and the mechanism of exosome action in mice. (B) Histological images of hematoxylin–eosin stain and Masson staining depicting osteocalcin in femurs treated with trimethylamine‐N‐oxide (TMAO) or PELNs. Scale bar, 200 µm. (C) Staining to observe the mineralization of human bone mesenchymal stem cells (hMSCs) incubated with TMAO or PELNs. (D) Alkaline phosphatase activity of hMSCs incubated with TMAO or PELNs. Reproduced with permission from reference^71^, Copyright© 2023, Elsevier.

*Pueraria lobata*, the dried root of the leguminous plant *Pueraria mirifica*, is an edible herb known for its medicinal and food properties in China. It contains active ingredients, such as flavonoids and Pueraria, which have various effects, including blood sugar regulation, blood lipid regulation, blood vessel protection, oxidative stress resistance, infection resistance, and tumor resistance.^68^, ^69^ *P. lobata*‐derived exosome‐like nanovesicles (PELNs) promote M2‐macrophage polarization, whereas *P. lobata* itself plays a significant role in regulating inflammatory responses in pathophysiological processes.^70^ Zhan et al.^71^ verified the effect of PELNs on the intestinal flora by constructing an ovariectomized (OVX) rat model to simulate osteoporosis (OP) (Figure 2A). Masson's trichrome staining showed that collagen fiber deposition was significantly reduced in the bone tissues of the trimethylamine‐N‐oxide (TMAO) group, and this inhibitory effect was reversed by PELNs (Figure 2B). They also^71^ confirmed that PELNs promoted the differentiation and mineralization of primary human bone mesenchymal stem cells (hMSCs) to treat OP (Figure 2C,D). Although this study did not directly target HIRI, its effect on intestinal flora provides a novel concept for the treatment of liver disease.^71^

#### 3.1.2 *Portulaca oleracea* L

**FIGURE 3:**
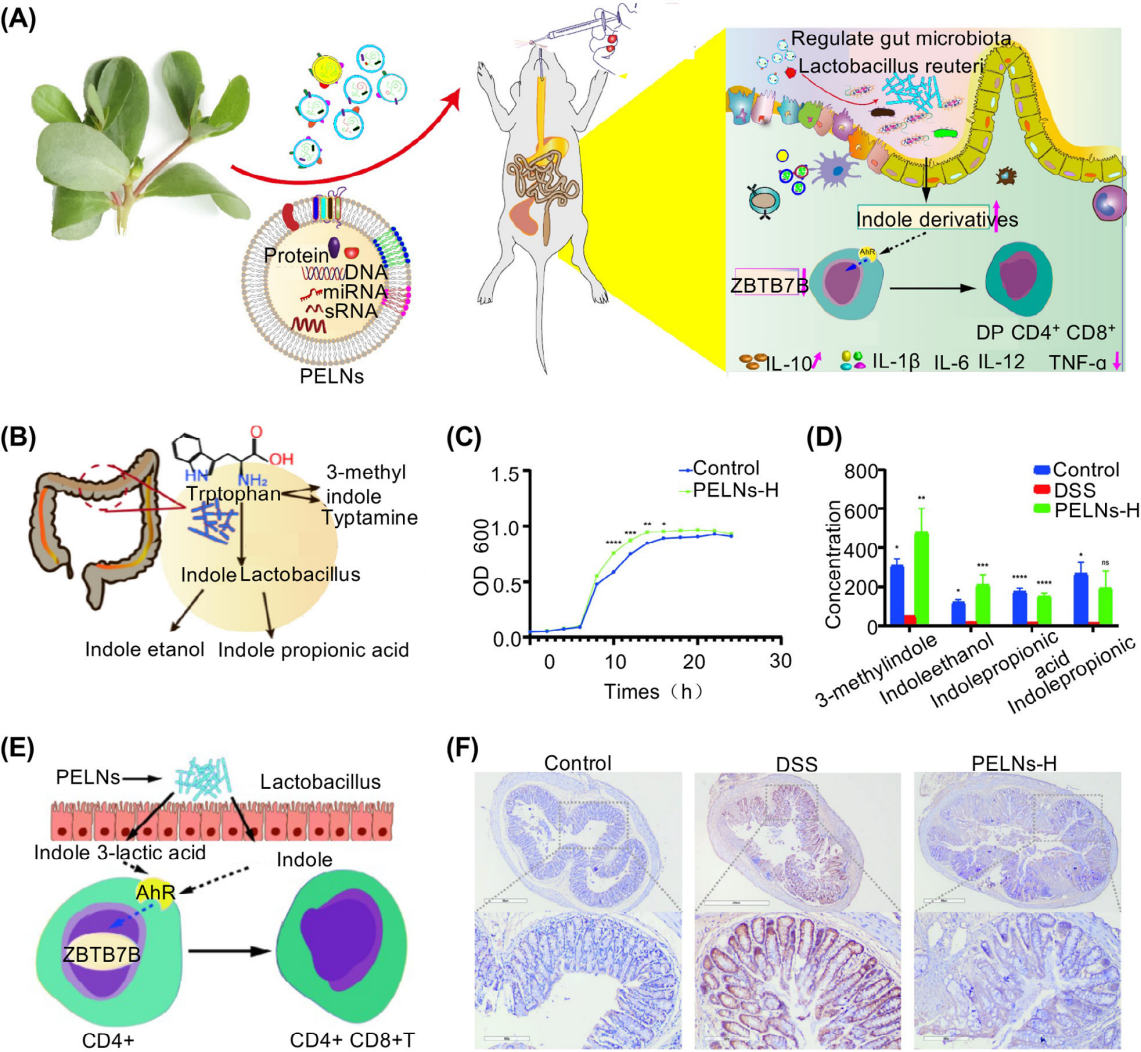
Edible *Portulaca oleracea* L.‐derived exosome‐like nanoparticles (PELNs) mitigate colitis. (A) Schematic of therapeutic effects of PELNs on ulcerative colitis. (B) Mechanisms for *Lactobacillus reuteri* metabolizing tryptophan into indole derivatives are represented schematically. (C‐D) PELNs increase the levels of indole derivatives. (E) Derivatives of *Lactobacillus reuteri* activate the aryl hydrocarbon receptor, thereby the reprogramming of conventional CD4+T cells into CD4+CD8+T cells. (F) PELNs significantly reduced the levels of Zbtb7b protein. Scale bar, 200 µm. Reproduced with permission from reference ^76^, Copyright© 2023, Nanobiotechnology.

*Portulaca oleracea* L. (PO) is a medicinal plant containing various active compounds, including polysaccharides, alkaloids, flavonoids, and fatty acids.^72^ It is recognized for its traditional medicinal properties, including analgesic effects and capacity to clear heat and remove toxins.^73^ Jang et al.^74^ focused on the anti‐inflammatory activity and extracted, isolated, and characterized the active substances from PO. The results revealed that the main anti‐inflammatory component of PO is derived from a less polar compound, petroleum ether. This compound, identified as stearic acid was further purified.^75^ Zhu et al.^76^ reported that PO‐derived exosome‐like nanoparticles (PELNs) could alleviate colitis induced by dextran sulfate sodium (DSS). This was achieved by promoting the expansion of double‐positive CD4+ and CD8+ T‐cells (Figure 3A). This mechanism involves the activation of the aryl hydrocarbon receptor by stimulating the growth of Lactobacillus and increasing the levels of indole derivatives. Consequently, the Zbtb7b levels were downregulated. (Figure 3B–F).^77^ These findings suggest that the oral administration of plant‐derived EVs is a targeted treatment for inflammatory bowel disease (IBD).^78^

#### 3.1.3 Tea

**FIGURE 4:**
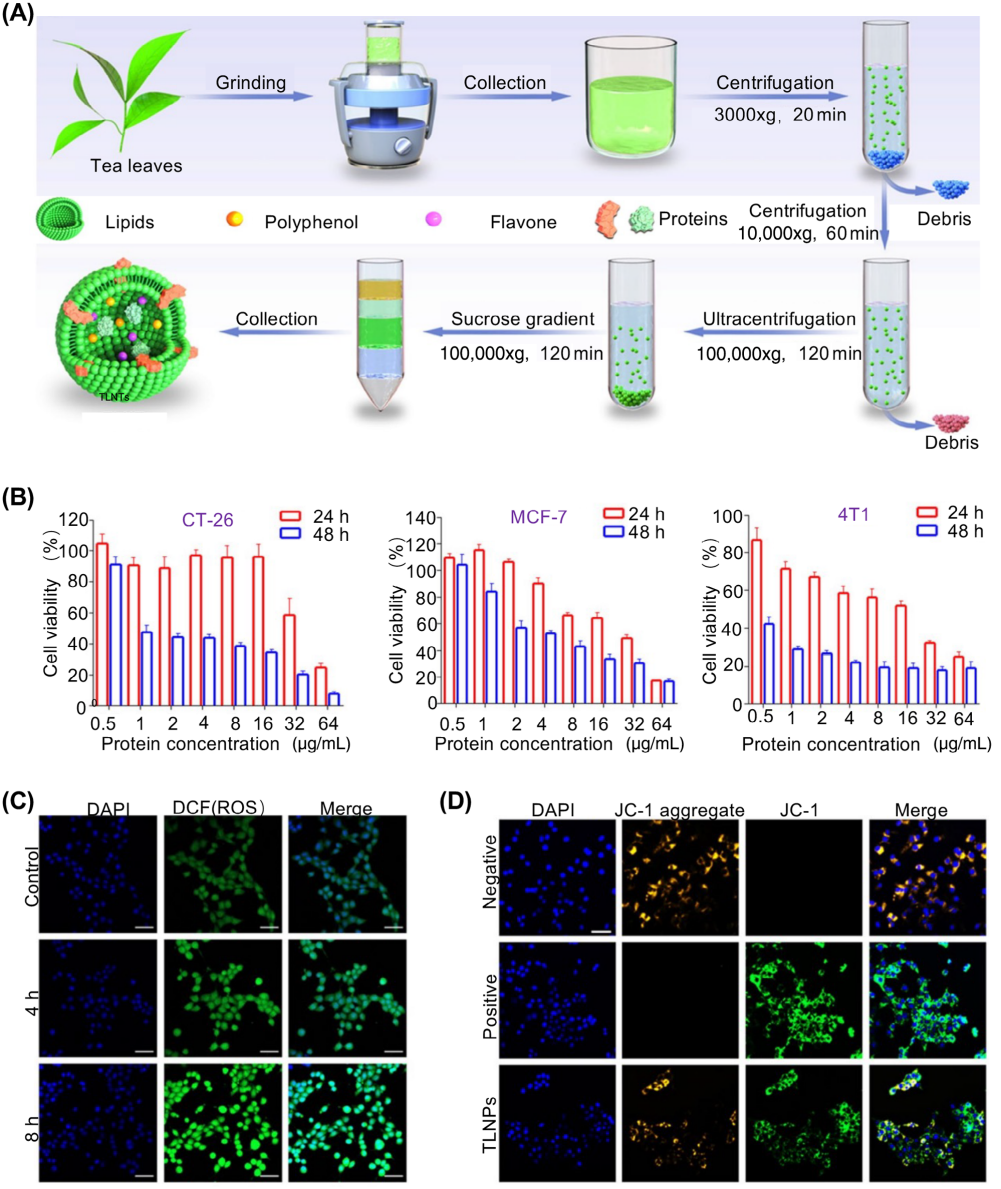
Extraction and purification process of nanotherapeutics derived from tea leaves (TLNTs), as well as their antitumor mechanism. (A) TLNTs are obtained through a process involving ultrahigh‐speed centrifugation and density gradient centrifugation, as depicted in the schematic representation. (B) Cytotoxicity of tea‐derived exosomes against CT‐26, MCF‐7,4T1 cells were determined after coculture for 24 and 48 h at different protein concentrations. (C) 2′−7′‐dichlorodihydrofluorescein diacetate (DCFH‐DA) stained images of 4T1 cells are captured after coincubation, providing insights into the effects of TLNTs. Scale bar, 50 µm. (D) Mitochondrial membrane potential. Scale bar, 50 µm. Reproduced with permission from reference ^80^, Copyright© 2023, Elsevier.

Tea, derived from the leaves and buds of tea trees, is an evergreen shrub commonly used to make tea.^79^ Exosome‐like nanotherapeutics derived from tea leaves (TLNTs) contain abundant polyphenols, flavonoids, functional proteins, and lipids. TLNTs were extracted from fresh tea leaves using a process involving differential centrifugation and subsequent ultracentrifugation to purify the TLNTs (Figure 4A).^80^ Upon coculture with TLNTs, the viability of CT‐26, MCF‐7, and 4T1 cells decreased with increasing TLNTs concentrations and longer incubation times (Figure 4B). By assessing the intracellular reactive oxygen species profile in the TLNT‐treated cells, we observed that the green fluorescent signal increased after TLNTs treatment and was predominantly localized within the entire cell. Thus, it was demonstrated that cytosolic polyphenols and flavonoids enhance oxidative stress in tumor cells (Figure 4C,D).^81^ Chen et al.^82^ provided new insights into the use of plant‐derived exosomes to inhibit extraintestinal tumors via the oral route and shed light on the therapeutic effects and potential mechanisms of tea, offering a theoretical foundation for clinical applications.

### 3.2 Modified plant‐derived EVs

The modification of plant‐derived EVs is a crucial aspect of EV‐based drug delivery systems. EVs offer significant advantages as drug delivery.^83^, ^84^ EVs typically exhibit high stability, prolonged circulating half‐life, and excellent safety profiles, ensuring the safe delivery of drugs to target cells.^85^ Moreover, EVs possess natural targeting capabilities with an overall small particle size of ∼100 nm, which enhances their targeting efficiency. Notably, the phospholipid bilayer structure of EVs improves the drug encapsulation rates, thereby enhancing drug stability.^86^ As natural carrier systems, EVs are characterized by low immunogenicity, high biocompatibility, and robust targeting abilities. Two primary pathways for drug loading into EVs have been identified: internal and surface modification. Internal modification techniques include electroporation, coincubation, sonication, freeze–thawing, and other methods for loading drugs into plant vesicles.^87^ Surface modification strategies include genetic engineering and chemical modification to enhance the drug delivery efficiency of EVs.

#### 3.2.1 Interior modification

Internal modification refers to the process of drug transfer into the EVs. This is primarily achieved by isolating and purifying EVs from plants, and then either coculturing the drug with EVs or loading the drug into EVs through sonication or electroporation.^88^ These methods ensure the effectiveness and stability of drugs. Internal modifications can be broadly categorized into two types: active incorporation by coculture and passive incorporation by sonication, extrusion, freeze–thawing, and electroporation.

Coculture is suitable for small‐molecule drugs with low cytotoxicity. Lipophilic chemicals such as curcumin can be successfully transfected into EVs using a coculture method. Passive incorporation, in contrast, involves the diffusion of the drug into EVs, mainly through concentration‐gradient differences.^89^, ^90^ This process is simple and preserves the integrity and functionality of EVs; however, it takes longer and has a lower drug loading rate.

The active incorporation approach uses tools to disrupt the EVs' membrane, allowing the drug to enter the interior. After successful loading, a constant‐temperature incubation was used to restore the membrane, followed by the removal of any free, unloaded drug. Electroporation involves the administration of a pulsed current into a specific solution to create gaps in the phospholipid bilayer of the EVs for drug loading. Ultrasound uses sound waves and homogenization probes to deform membranes and load drugs. In the extrusion method, molecules are passed through a 300 nm‐sized membrane. Freeze–thawing requires repeated freezing of the drug mixture in liquid nitrogen to load the drug.

Haney et al.^91^ used a variety of methods to load peroxidase inside EVs, such as sonication, extrusion, and freeze–thaw cycles. The findings revealed that The first two methods achieve loading rates of ∼30%. Among these methods, sonication and extrusion demonstrated higher drug loading and greater loading capacity than other approaches. The active conjugation loading process is convenient and easy to regulate; however, it may compromise the integrity of EVs, which is not ideal for subsequent experiments.^92^, ^93^

#### 3.2.2 Surface modification

In contrast, chemical modification involves directly modifying isolated and purified EVs. This can be achieved through coupling reactions or lipid assembly to display various ligands for more specific delivery to the target cells.^94^, ^95^ Coupling reactions modify the surface proteins of EVs through covalent binding; however, the complexity of the EVs’ surface can slow the reaction rate. Chemical modifications can also damage the surface structures of EVs. Furthermore, lipid molecules can penetrate the hydrophobic layers of EVs, thereby exposing their hydrophilic portions. This lipid‐mediated approach may lead to EV toxicity. For instance, in a study by Tian et al.,^96^ the c(RGDyK) peptide was chemically modified on the surface of EVs. The peptide was injected into C57BL/6 mice via tail vein. These results suggest that chemically coupled exosomes effectively treat ischemic stroke by strongly inhibiting the inflammatory response and apoptosis in the lesion area. Another application of chemical modifications is targeting cancer cells with paclitaxel‐modified AS1411‐cholEVs, which provide a broad delivery platform for cancer therapy.

## 4 DELIVERY STRATEGIES

**FIGURE 5:**
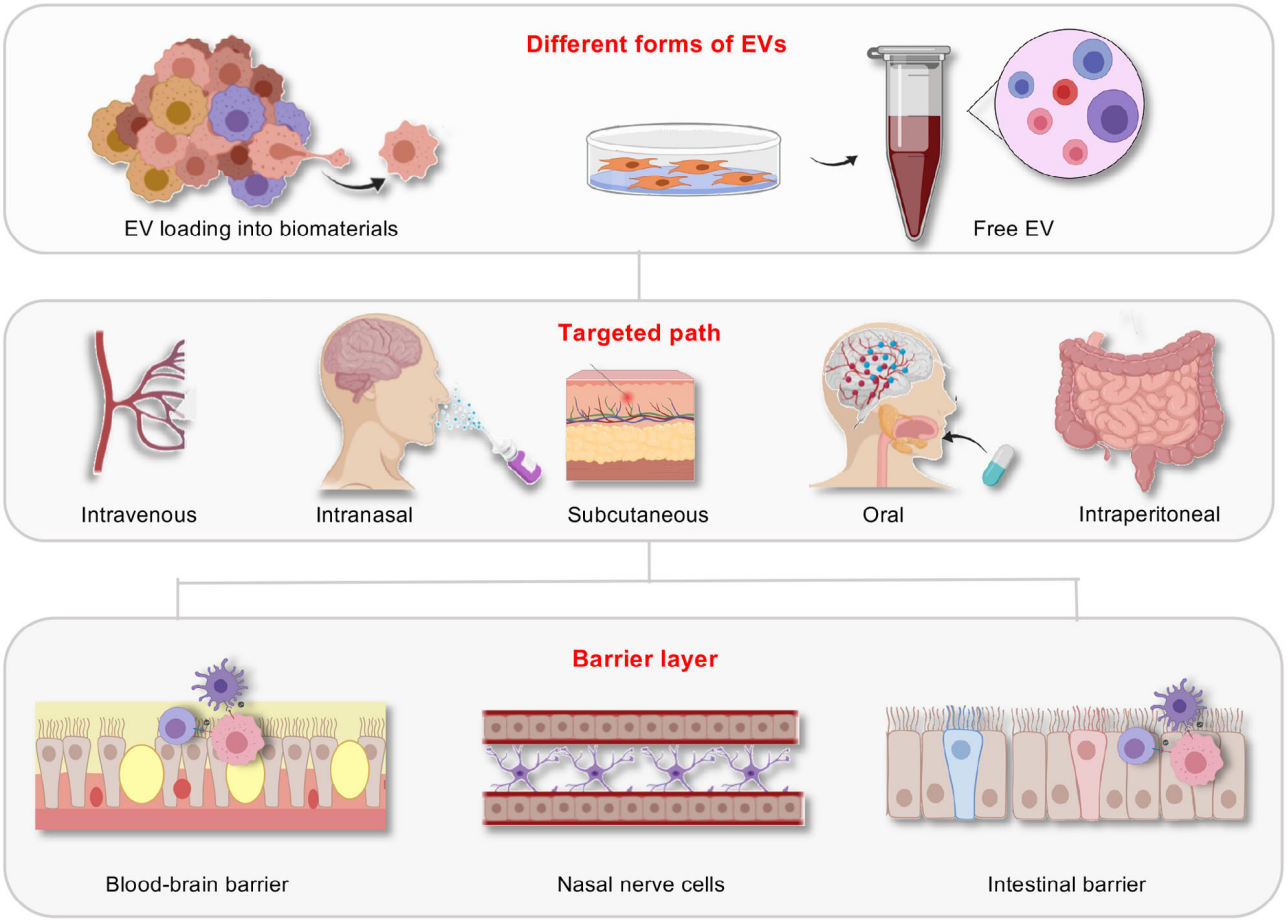
Extracellular vesicles (EVs) enter the body in various delivery modes. Efficacy and targeting of EVs vary depending on their formulation and delivery method. The blood–brain barrier can be crossed through intravenous and nasal administration, while the intestinal barrier can be overcome through intraperitoneal administration. In the case of the blood–brain barrier, EVs may also be internalized by structures such as the trigeminal nerve and transported retrogradely through nerve cell axons (created by MedPeer).

EVs have garnered increasing attention in clinical medicine as messengers for intercellular communication and carriers for targeted delivery of RNA and drugs.^97^, ^98^ However, despite numerous researchers utilizing EVs for drug delivery, challenges remain in achieving successful targeting of cells in vivo, as well as ensuring the content and purity of EVs upon reaching the target cells. To accurately assess their therapeutic potential in clinical settings, it is crucial to explore reliable delivery strategies that ensure the precise targeting of diseases while maximizing efficacy prior to clinical application.^99^, ^100^, ^101^ Therefore, we compiled a list of key delivery modalities for clinical applications, including intravenous, oral, intraperitoneal, and subcutaneous administration and further explored and compared the dosages and advantages of these modalities for improved biomedical applications (Figure 5 and Table 1).^102^, ^103^, ^104^

**TABLE 1:** Types of plant‐derived extracellular vesicle administration.

Administration route	Disadvantages		Tissue distribution in animals	References
	Advantages	Disadvantages		
Intravenous injection	Highest bioavailability of all delivery modes Absence of absorption and first‐pass metabolism barriers	Repeated injections over an extended period of time must be avoided	Mainly in the liver and spleen	^105^
Oral administration	Due to first‐pass effect bioavailability of only 20−30% Better patient compliance	Causes peaks and valleys and increases side effects	Mainly in the gastrointestinal tract	^106^
Intraperitoneal injection	Bioavailability up to 80% large absorption area and high absorption capacity	Highly irritating Difficult to puncture the abdominal wall	Gastrointestinal tract and lymph nodes	^107^
Subcutaneous injection	Bioavailability of 40% mostly used for localized administration	Limited to small doses of drugs with Strong pharmacological effects	Mainly in injection sites or lymph nodes	^108^

### 4.1 Intravenous injection

Intravenous injection is the method of delivering medication directly into a vein using a syringe.^109^ It is considered the most efficient mode of drug delivery, offering advantages, such as high bioavailability and the ability to administer a single injection or continuous drip without the need for solvent uptake or metabolism.^110^ In mice and rats, three tail veins are commonly used, one on each side of the tail and the other on the dorsal side. These veins are preferred for injections because of their thin cuticles and ease of fixation.^111^ Intravenously administered EVs reach the liver, spleen, and other sites. Further, intravenous injection is generally advantageous because it bypasses the first‐pass effect of hepatic metabolism, resulting in high bioavailability. Consequently, the intravenous administration of EVs is frequently employed in tumor treatment and represents a novel approach for in vivo therapy.^112^, ^113^

Li et al.^114^ successfully isolated EVs from ginger, enabling their use as a delivery system for siRNA to target cells through intravenous injection every 2 days for 2 weeks. These results indicated that ginger, along with other edible plants, can produce significant quantities of EVs and demonstrate tumor suppression in a human oral epidermal carcinoma cell‐derived xenograft mcodel.^114^ Furthermore, intravenously delivered EVs serve as effective vaccines to induce antitumor immunity in mice. In an initial study on intravenously administered EVs, they ameliorated acute kidney injury in mice. Several other studies involving systemic intravenous injection have successfully enhanced various brain conditions, suggesting that EVs injected into systemic circulation can cross the blood–brain barrier.^115^ In summary, intravenous drug delivery strategies can target tumors and sites related to the blood–brain barrier, offering new insights for the treatment of clinical diseases.

### 4.2 Oral administration

Oral drug delivery refers to the absorption of medications into the bloodstream through the gastrointestinal tract after oral intake. This allows the drug to reach local or systemic tissues via the bloodstream, effectively treating targeted disease.^116^, ^117^ However, the proteins and nucleic acids within exosomes are vulnerable to degradation in the gastrointestinal environment, making oral drug delivery challenging.^63^, ^118^ Some studies have utilized the layer‐by‐layer self‐assembly technology to create multilayer membrane structures, protect encapsulated substances, and maintain metabolic activities and functions.^119^, ^120^

Ou et al.^121^ successfully isolated exosome‐like nanovesicles derived from the leaves of *Catharanthus roseus* (L.) Don, referred to as CLDENs. Mice were administered 60 mg/kg of CLDENs via tail vein injection, intraperitoneally, and orally, and their in vivo distribution was analyzed.^121^, ^122^ The results indicated that the drug was primarily distributed in the stomachs of mice within 12 h. Based on these findings, we hypothesized that CLDENs are stable and resilient in the gastric fluid, exhibiting a strong capacity for gastric absorption. After intraperitoneal injection, it remains mainly in the immune organs, including the thymus, spleen, liver, and kidneys. Intraperitoneal injection results in broader systemic absorption than tail vein injection groupc.^123^, ^124^ In conclusion, CLDENs can remain in the gastrointestinal tract for an extended period after oral administration owing to their robust resistance to gastric acid. Therefore, the use of orally administered therapy in clinical trials may be an effective strategy to significantly enhance patient compliance.

### 4.3 Intraperitoneal injection

Intraperitoneal injection involves injecting drugs into the extra plasma membrane and into the gastrointestinal tract of animals.^125^, ^126^ It is commonly used for drug delivery owing to its ease of use and rapid absorption. Intraperitoneal injection is typically used for drugs that are poorly absorbed in the gastrointestinal tract or have low absorption efficiency. It is also used in experiments involving anesthesia in mice.^127^ Compared with intravenous injection, transperitoneal administration leads to slower drug absorption, but allows for a larger volume of drug to be injected, which is beneficial for smaller experimental animals.^128^ Following intraperitoneal injection, exosomes are distributed to various organs such as the liver, spleen, lymph nodes, and gastrointestinal tract in mice. In rats, the signal was weaker in the liver than in the spleen, and the exosomes were primarily distributed in the abdominal and cervical lymph nodes.^129^

Intravenously injected plant‐derived EVs can enter the circulation directly and rapidly and are distributed throughout the body's organs. However, the liver and spleen absorb most of the therapeutic effects of the intravenous injections. In contrast, EVs administered intraperitoneally require a significant amount of time to circulate in the bloodstream, allowing access to other tissues in the body. Additionally, intraperitoneal injection provides visceral fat and intestines with greater opportunities to absorb EVs, owing to the ability of exosomes to cross basement membranes and physiological barriers. Li et al.^130^ investigated the efficacy of exosome‐like nanovesicles derived from *Hypericum perforatum* (HPExos) in treating obesity‐related adipose tissue and metabolic disorders. They injected HPExos intraperitoneally into C57BL/6J obese mice at a dose of 20 µg/mL and then imaged the mice in vivo 4 h after administration.^130^ The results revealed that the fluorescence signal was significantly stronger in the intestinal adipose tissue of obese mice than in other organs, indicating that intraperitoneal injection is a noninvasive method for targeting adipose tissue in the treatment of obesity and metabolic disorders. In conclusion, intraperitoneal injections are suitable for the treatment of intestinal and metabolic disorders.^131^, ^132^

### 4.4 Subcutaneous injection

Subcutaneous injection is a method of administering medication by injecting it into a layer of connective tissue located beneath the skin. This allows the medication to be absorbed into the bloodstream through capillaries and lymphatic vessels. This method is commonly employed for drug delivery.^133^ Subcutaneous injections are typically administered to areas with thin skin, loose subcutaneous tissue, and few blood vessels, such as the back of the neck, armpits, or inner thighs. It is suitable for easily dissolved, nonirritating drugs as well as bacterial vaccines, vaccines, and cells. Subcutaneous injection helps avoid the direct entry of exosomes into circulation, reducing hepatic and splenic clearance. However, significant exosome aggregation may occur in the pancreas and digestive tract. In mice and rats, after the subcutaneous injection of exosomes, in vivo bioluminescence imaging indicated that the radioactive signal remained primarily at the injection site, with a small signal observed in the abdomen surrounding the gastrointestinal tract.^134^ In nonhuman primates, following subcutaneous injection, exosome signals are mainly clustered in the ipsilateral inguinal lymph nodes, with some residual signals observed in the gastrointestinal tract.

Subcutaneous injections of plant‐derived EVs are commonly used for skin wound repair. The promotion of neovascularization, facilitation of collagen synthesis and remodeling, and modulation of local inflammation are the key therapeutic advantages.^135^ Moreover, plant‐derived EVs are easier to produce, have high stability, and low immunogenicity. After subcutaneous injection, EVs penetrate deep into the stratum corneum through local transdermal absorption via transcellular and intercellular pathways, allowing for diffusion, absorption, and onset of efficacy.^136^ In an in vitro study, broccoli exosomes were found to have good morphological integrity and a high encapsulation rate. Specifically, it demonstrated good stratum corneum penetration using a subcutaneous delivery strategy.^137^ Therefore, the subcutaneous injection strategy is well targeted and suitable for the clinical treatment of local trauma and medical cosmetology.

## 5 BIOMEDICAL APPLICATION

The mechanism of action of plant‐derived EVs primarily involves anti‐inflammatory, antioxidant, antitumor, and the maintenance of gut microbial functions. Garlic exosomes effectively treat DSS‐induced inflammation in the mouse colon by suppressing the production of proinflammatory factors and reshaping the gut flora.^138^, ^139^ Lemon‐derived EVs inhibit the progression of chronic granulocytic leukemia by targeting tumor sites and preventing apoptosis.^140^ Recently, artemisinin‐derived EVs exhibited immunomodulatory effects by activating the cGAS–STING pathway in a mouse lung cancer model.^141^ Although significant progress has been made toward understanding the therapeutic potential of EVs, their specific mechanisms of action and efficacy remain largely unknown. Therefore, further research focusing on the diverse functions of EVs derived from various plant sources and their biomedical applications is crucial to advance our understanding of this field.

### 5.1 Anti‐inflammatory

Inflammation is a defense response to abnormal stimuli caused by pathogenic bacteria, cellular damage, or ischemia. This crucial process helps combat pathogens and facilitates tissue repair and regeneration. However, severe inflammation and the prolonged absence of anti‐inflammatory interventions can result in varying degrees of irreversible damage, potentially leading to the development of complex diseases, such as cancer, cardiovascular issues, and neurological disorders.^142^, ^143^ Currently, the main drugs used clinically to control inflammation include nonsteroidal anti‐inflammatory drugs, such as aspirin, and glucocorticoids, such as dexamethasone. However, these conventional anti‐inflammatory medications are associated with the risk of developing resistance.^144^ Therefore, there is a pressing need to explore and develop anti‐inflammatory agents derived from natural plant sources as potential alternatives to address these challenges.

Wu et al.^145^ used gradient filtration in conjunction with high‐speed centrifugation to isolate EVs from *P. mirifica*. Their study revealed that the uptake of *P. mirifica*‐derived EVs significantly reduced the expression of proinflammatory factors associated with M1‐like macrophages. In a study by Zhang et al.,^146^ colonic inflammation was induced in 6−8 week C57BL/6 mice using DSS. Ginger‐derived exosomes obtained through ultra‐high‐speed centrifugation were orally administered to mice in the model, leading to their in vivo distribution in colonic tissues after uptake by intestinal epithelial cells and macrophages. These findings demonstrated that ginger‐derived EVs reduced the expression of inflammatory factors. Additionally, these exosomes promoted the repair of damaged mucosa, highlighting their potential as novel anti‐inflammatory therapeutic agents, particularly in the context of *P. lobata*‐derived EVs.

### 5.2 Antioxidant

Oxidative stress arises from the inflammatory infiltration of neutrophils due to an imbalance in reactive oxygen species within the body. This can result in cell damage and death, contributing to the development of various diseases. Although many plants possess inherent antioxidant properties, clinical antioxidants such as polyphenolic compounds, vitamin C, and vitamin E are often derived from plant sources.^147^, ^148^ However, the purification efficiency of these natural antioxidants is limited, their purification processes are not well defined, and they are rapidly metabolized in vivo.^149^ Therefore, there is a critical need to explore methods for isolating more potent antioxidants from plant‐derived EVs to combat excessive reactive oxygen species and address disease conditions effectively.^150^

Kim et al.^151^ successfully isolated high concentrations of EVs from carrots using size‐exclusion chromatography combined with ultrafiltration. They demonstrated low cytotoxicity of these EVs through in vitro assays, demonstrating their ability to inhibit oxidative stress and apoptosis in human neuroblastoma cells of cardiac origin derived from H9C2 embryonic rats. These findings suggest that carrot‐derived EVs hold promise as potential therapeutic agents for conditions such as myocardial infarction and Parkinson's disease.^151^ Tuong et al.^152^ extracted EVs from blueberries by successive low‐speed centrifugation in the presence of class I chitinases. Blueberry‐derived EVs have been found to downregulate proinflammatory factors and total glutathione, exhibiting antioxidant properties.^152^ Furthermore, these EVs demonstrated high drug‐carrying capacity, with the ability to load up to 80% curcumin, further supporting their potential as effective carriers of therapeutic compounds.

### 5.3 Antitumor

Clinical treatment of tumors faces several challenges, including drug resistance, lack of targeting, and severe side effects. Current treatment modalities for tumors include interventional therapy, immunotherapy, chemotherapy, and radiotherapy.^153^, ^154^ However, these approaches are constrained by factors, such as individual patient variations, differing levels of tolerance, surgical constraints, drug toxicity, and economic considerations.^155^ As a result, the prevention and treatment of tumors remain significant challenges for humanity. Plant‐derived EVs offer advantages, such as low toxicity and easy availability, making them promising candidates for exploring novel approaches to tumor treatment.^156^ Given the potential benefits of plant‐derived EVs, the study and development of strategies to use these natural resources to address the complexities of tumor management is critical.

In a study conducted by Liu et al.,^157^ EVs obtained from the isolation and purification of artemisinin were administered intraperitoneally to adult C57 mice with lung cancer. The research findings highlighted that artemisinin‐derived mitochondrial DNA played a pivotal role as a major effector molecule in triggering the cGAS–STING pathway.^157^ This mechanism leads to remodeling of the tumor microenvironment, reprogramming of tumor‐associated macrophages, and conversion of tumor‐promoting cells into an antitumor phenotype. Consequently, antitumor effects were observed, which enhanced immunity. Huang et al.^158^ isolated EVs from Centella asiatica by high‐speed centrifugation. The morphology, particle size, and protein markers of exosome‐like Centella asiatica‐derived nanovesicles were characterized using TEM, and their in vitro antitumor effects were assessed. This study revealed the presence of numerous cancer‐targeting miRNAs in these EVs, with potential effects on the proliferation of HepG2 cells through metabolic pathways such as amino acid and lipid synthesis. Collectively, these findings suggest that EVs derived from Centella asiatica hold promise as novel applications of plant‐derived nanomedicines in cancer immunotherapy.

### 5.4 Maintenance of gut microbial

The gut microbiota maintains host physiology and can be influenced by dietary interventions such as food choices. However, disruptions in the intestinal mucosa caused by an unhealthy diet or certain diseases can lead to microbiota dysbiosis, necessitating clinical intervention with medications. Plant‐derived EVs, particularly those from vegetables rich in nucleic acids, such as RNA, have shown promising therapeutic effects in modulating the gut microbiota and enhancing host physiological functions upon absorption in the intestine.^159^, ^160^ Plant‐derived EVs strengthen the intestinal barrier, improve immune responses, and ameliorate metabolic disorders associated with alterations in gut microbiota.^161^, ^162^ Plant‐derived EVs positively affect the gut and overall health by regulating the composition or functions of the gut microbiota. Their ability to modulate the intestinal flora and enhance host physiology highlights the therapeutic potential of plant‐derived EVs in promoting gut health and addressing related disorders.

Pang et al.^163^ investigated the effects of EVs isolated from kidney beans on high‐fat diet‐induced obesity. Orally administered kidney bean‐derived EVs demonstrated significant reductions in body and liver weights and improved obesity levels in obese rats.^164^ These findings indicate that kidney bean‐derived EVs enhance the diversity of the intestinal flora, mitigate diet‐associated obesity by improving gut microbiota, and increase short‐chain fatty acid production. Constipation, a common gastrointestinal issue that can impact quality of life and lead to other health concerns, has been targeted for treatment through the modulation of gut microbiota. Duan et al.^165^ explored the potential of broccoli‐derived EVs in alleviating constipation in a mouse model of loperamide‐induced constipation. Oral administration of broccoli‐derived EVs effectively increases bowel movements and accelerates intestinal peristalsis in constipated mice. The mechanism underlying these effects involves the modulation of the gut microbiota and microbial tryptophan metabolism, ultimately relieving constipation. These studies highlight that plant‐derived EVs are stabilized in the gut and influence the composition of the intestinal flora, offering promising therapeutic strategies for addressing conditions such as obesity and constipation through modulation of the gut microbiota and metabolic pathways.

## 6 THERAPEUTIC POTENTIAL IN SPECIFIC DISEASES

Significant advances have been made in research on the efficacy of plant‐derived EVs, revealing their modulatory effects in various fields, including antitumor therapy, immunomodulation, intestinal diseases, and regenerative medicine. These plant‐derived EVs have demonstrated considerable potential and their therapeutic effects have been validated in multiple studies.^166^, ^167^ Despite these promising findings, clinical trials have predominantly focused on mammalian EVs. Currently, there are only three registered clinical trials involving plant‐derived exosomes: exosomes derived from grapes (referred to as “grape extract,” NCT01668849), ginger, and aloe vera (referred to as “exosomes,” NCT03493984).^168^, ^169^ Specifically, grape‐derived exosomes are being investigated for the treatment of radiotherapy‐induced oral mucositis, whereas ginger‐and aloe vera‐derived exosomes are being studied for their effects on polycystic ovary syndrome‐induced insulin resistance and chronic inflammation. Given this background, we aimed to reveal the pharmacological activities of several plant‐derived EVs in disease treatment and explore their underlying mechanisms, particularly in preclinical animal models (Table 2).^170^ We hope to provide new insights and ideas for future clinical trials of plant‐derived EVs and their potential therapeutic applications.^171^

**TABLE 2:** Preclinical trial of plant‐derived extracellular vesicles and its mechanism.

Type of diseases	Plant sources	Separation method	Animal model	Dose	Mechanism of action	References
Hepatocellul‐ar carcinoma	Asparagus cochinchinensis	Differential centrifugation	Male BALB/c mice	200 mg/kg for seven doses	Internalized by tumor cells, extending blood circulation duration and augmenting the concentration at the tumor site.	^172^
Triple‐negative breast cancer	Brucea javanica d	High speed centrifugation	Female BALB/c mice	6 mg/kg every 3 days	Effectively slowing down the growth and spread of 4T1 cells by modulating the PI3K/Akt/mTOR signaling pathway and enhancing ROS/caspase‐mediated apoptosis.	^173^
Glioma	Ginseng	High speed centrifugation	Male Wistar rats	1 mL of 2 mg solution for seven doses	Regulating protumoral cytokines, stimulating T cell production, and inhibiting regulatory T cells within the tumor microenvironment.	^174^
Ulcerative colitis	Turmeric	Ultracentrifugation sucrose gradient centrifugation	The FVB/NJ female mice	3 mg/dose	Controlling the expression of proinflammatory cytokines such as TNF‐α, IL‐6, and IL‐1β, as well as the antioxidant gene HO‐1.	^175^
Type 2 diabetes	Garlic	Ultracentrifugation	C57BL/6 mice	1 × 10^10^ particles/20 g	Elevating the levels of OMV Amuc‐1100, P9, and phosphatidylcholines.	^139^
SARS‐CoV‐2	Peeled Hawaiian ginger roots	Ultracentrifugation overnight	The mouse C57BL/6	5 mg/kg/day	miRNA inhibits SARS‐CoV‐2‐induced lung inflammation and viral replication.	^16^
Spinal cord injury	Lycium barbarum L.	Sucrose density gradient centrifugation	The SD rats (male, 200 g)	3D‐printed bionic scaffold	Modulating the inflammatory response post spinal cord injuries, promoting the repair of damaged axons, and ultimately enhancing neurological function.	^176^
Leukemic	Grapefruit	Ultracentrifugation	Newly diagnosed de novo AML patients	1 × 10^12^ administered every 2 days	Elevating ROS levels in AML blast cells and U937 cells without affecting ROS levels in normal cells, similar to the impact of 2 mM ascorbic acid.	^177^
Alcoholic intoxication	Pueraria lobata root	Differential centrifugation	C57BL/6J mice	10 mg/(kg bw)	Preventing ferroptosis by inhibiting the reduction of glutathione peroxidase 4 and reduced glutathione suppressing the elevation of acyl‐CoA synthetase long chain family member 4.	^146^

### 6.1 Cancer therapy

Plant‐derived EVs are abundant medicinal components that exhibit potent anticancer effects through various mechanisms. They can influence intercellular communication, improve the tumor microenvironment, inhibit the secretion of tumor‐derived exosomes, and enhance the sensitivity of drug‐resistant bacteria to therapeutic agents.^178^, ^179^ These EVs often play a critical role in reducing cancer cell resistance to chemotherapy and mitigating adverse reactions associated with cancer treatment by engaging in multiple signaling pathways.^180^, ^181^ Their metastable nature allows them to act as carriers, transfer therapeutic components to target cells, and exert antitumor effects. Notably, the pharmacological activities of EVs can vary significantly depending on the plant source, resulting in diverse antitumor mechanisms. This variability underscores the potential for tailored therapeutic applications of plant‐derived EVs in oncology because different biocomponents may preferentially target specific cancer types or resistance mechanisms. Continued research on these pathways and mechanisms is vital to optimize the clinical use of plant‐derived EVs in cancer treatment.

*Asparagus cochinchinensis* (Lour.) Merr. (ACNVS) is a well‐known medicinal plant recognized for its various therapeutic effects, including anticancer, antioxidant, and anti‐infective properties.^182^ In a study conducted by Zhang et al.,^172^ exosome‐like nanovesicles derived from ACNVs were isolated using ultra‐high‐speed centrifugation combined with sucrose density gradient centrifugation. This study demonstrates the antiproliferative role of ACNVs in hepatoma carcinoma cells and explores their apoptosis‐inducing mechanisms using adult male BALB/c mice as experimental subjects.^183^ These findings reveal that ACNVs can be internalized into tumor cells primarily through phagocytosis. Once in the bloodstream, these nanovesicles enhance their circulation time by blocking scavenger receptors or modifying them with PEG. This modification facilitates the accumulation of ACNVs at the cancer sites, thereby improving their therapeutic efficacy. Additionally, PEGylation of ACNVs offers a significant advantage as it minimizes adverse reactions while effectively targeting cancer cells. By optimizing the pharmacokinetic profile of ACNVs via PEGylation, this study expands the potential biomedical applications of medicinal plant‐derived EVs and paves the way for novel therapeutic strategies for cancer treatment. This study highlights the promising role of ACNVs in the development of safer and more effective cancer therapies from natural sources.

**FIGURE 6:**
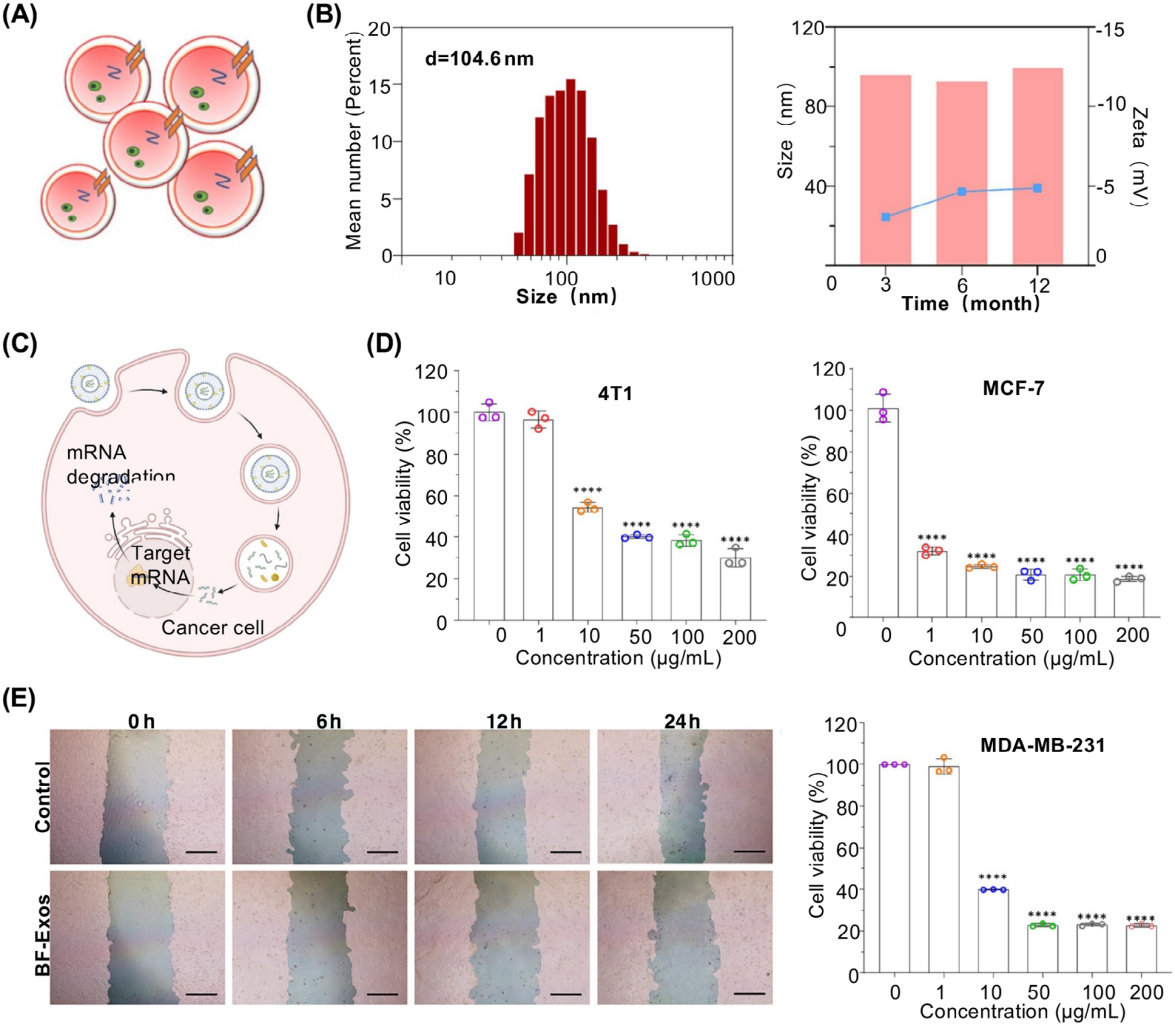
Strategies for treating 4T1 breast cancer with BF‐Exos. (A) Morphology of BF‐Exos. (B) Characterization of BF‐Exos. (C) BF‐Exos containing miRNA targets pathways that degrade mRNAs. (D) Inhibiting 4T1 cell growth and promoting apoptosis of cancer cells. (E) Quantitative migration of BF‐Exos‐treated 4T1 cells. Scale bar, 400 µm. Reproduced with permission from reference ^173^, Copyright© 2024, Elsevier.

Yan et al.^173^ conducted a study using high‐speed centrifugation to purify exosome‐like nanovesicles derived from Blumea javanica (BF‐Exos) (Figure 6A,B). This study employed a BALB/c mouse model of 4T1 breast cancer, specifically 6‐week‐old female mice. Mechanistic analyses revealed that BF‐Exos could effectively deliver certain functional microRNAs (miRNAs) to 4T1 cells, which are typically underexpressed in tumors (Figure 6C). These miRNAs regulated the PI3K/Akt/mTOR signaling pathway and inhibited the development of 4T1 cells, thereby suppressing tumorigenesis (Figure 6D,E). Additionally, a study highlighted that BF‐Exos can inhibit and regulate vascular endothelial growth factor. By targeting both tumor growth and angiogenesis, BF‐Exos represent a multifaceted approach to combat breast cancer. In summary, the findings of this study demonstrated that BF‐Exos serve as a novel delivery platform for phytotherapeutic agents, offering promising avenues for the clinical treatment of 4T1 breast cancer. Furthermore, this study explored the unique efficacies of natural plant compounds and elucidated their mechanisms of action in disease treatment through experimental validation, thereby expanding the research on plant‐derived EVs. This study underscores the potential of utilizing botanical resources to develop innovative strategies for cancer therapy.

### 6.2 Ischemia–reperfusion injury

IRI is a significant tissue injury caused by transplant‐induced ischemia that occurs when blood supply is restored and is commonly seen in conditions affecting the liver, myocardium, and brain. This type of injury can lead to inflammation and further tissue damage, which complicates recovery and treatment outcomes.^184^, ^185^ EVs have demonstrated therapeutic potential in mitigating the effects of IRI.^186^ Although mammalian EVs have shown promise, they are often associated with side effects and can be time consuming to produce.^187^ This has led to increasing interest in plant‐derived EVs, which offer advantages such as low immunogenicity and more efficient production processes. Recent studies have highlighted the potential of plant‐derived EVs to alleviate inflammation associated with IRI.^188^, ^189^ Successful outcomes have been reported in preclinical animal models, demonstrating the ability of EVs to modulate inflammatory responses and promote tissue repair after IRI. Despite these promising results, none of these findings have been transitioned to clinical practice, which limits their application in treating patients. To bridge this gap, it is essential to conduct preclinical animal studies investigating the therapeutic effects of plant‐derived EVs on IRI.^190^, ^191^ By analyzing these cases, we aimed to provide a theoretical foundation for future clinical applications by highlighting the mechanisms of action, efficacy, and safety profiles of plant‐derived EVs. This study could pave the way for innovative treatment strategies that harness the therapeutic potential of plant‐derived materials to manage IRI in clinical settings.

Panax notoginseng (PDN) is a widely recognized medicinal plant known for its beneficial effects in the treatment of IRIs, particularly acute ischemic stroke. Saponins derived from PDNs can effectively enhance neurological function and exhibit antiplatelet and anticoagulant properties. Li et al.^192^ conducted a study on exosome‐like nanoparticles derived from PDNs and evaluated their therapeutic potential in male Sprague–Dawley rats. These findings revealed that PDNs could directly enter the brain through the bloodstream, significantly reducing the size of the cerebral infarction and improving the behavioral outcomes of affected animals following IRI. Moreover, we identified a key mechanism by which PDNs exert their protective effects by modulating the inflammatory response associated with cerebral ischemia/reperfusion. Specifically, PDNs have been shown to promote the transition from an M1 macrophage phenotype to an M2 phenotype, thereby reducing overall inflammation. Furthermore, PDNs contain lipids that mediate the inhibitory inflammatory effects of the therapy. These lipids activate the PI3K/Akt signaling pathway, which is key to modulating inflammation. These results indicate that PDNs are prospective clinical candidates for the treatment of cerebral IRI. Their ability to enhance neurological function, reduce infarct size, and regulate inflammatory responses highlights the role of plant‐derived EVs in the management of complex neurovascular conditions. Further studies and clinical trials are necessary to validate these findings and fully explore the applicability of PDNs in clinical settings

Flavonoids found in medicinal plants have demonstrated the capacity to enhance cardiovascular health, prompting their use in preclinical studies as potential treatments for cardiovascular diseases. Lu et al.^190^ isolated EVs from flavonoids that showed promise in mitigating IRI. Specifically, nanovesicles derived from *Exocarpium citri grandis* (ENVs) have been identified to possess antioxidant properties capable of ameliorating IRI in the early phases of heart transplantation. In this study, MSCs were used to create membrane‐derived nanovesicles, which were then fused with ENVs and loaded with rapamycin, an immunosuppressive drug. This resulted in a hybrid drug delivery system that combined animal‐ and plant‐derived EVs. Rapamycin‐loaded EVs protect the heart from IRI by targeted delivery to the cardiac transplantation site. Importantly, ENVs polarize Ly6C^+^Ly6G^−^ inflammatory cells towards the M2‐type, which is associated with anti‐inflammatory and tissue repair functions. This shift in macrophage polarization contributes to the prolonged survival of cardiac grafts and is achieved without inducing therapeutic toxicity. These findings introduce a groundbreaking concept for the clinical management of early IRI after organ transplantation.^193^

### 6.3 Wound

Wound healing is typically a self‐regulating process; however, certain individuals suffer from chronic nonhealing wounds or abnormal scarring due to autoimmune disorders. This has led to an increasing demand for innovative therapeutic agents capable of accelerating the wound healing process.^194^ Plant‐derived EVs have emerged as promising treatment modalities for wound healing and tissue repair. These EVs are enriched with bioactive molecules, including growth factors and miRNAs, which are believed to facilitate the intercellular communication essential for tissue regeneration.^195^, ^196^ The role of EV‐based signaling is particularly crucial throughout the wound healing process and encompasses four key phases: wound closure, re‐epithelialization, neurogenesis, and angiogenesis. This underscores the potential for leveraging the innate healing properties of plants to stimulate rapid wound healing. Studies have demonstrated the efficacy of medicinal plant extracts with antihemorrhagic properties in wound healing.^197^ Plants have several applications in traditional medicine because of their wound‐healing benefits.^198^

Tan et al.^199^ successfully isolated exosomes from medicinal plant ginseng GExos using an ultrafast separation technique. GExos have demonstrated remarkable potential as nanomaterials for treating complex skin ulcers associated with diabetes. The study showed that GExos, when transferred to endothelial cells, enhanced their proliferation, migration, and remodeling under high glucose conditions. This is achieved by mitigating the oxidative stress‐induced reprogramming of glycolysis and promoting anaerobic glycolysis. To evaluate the in vivo therapeutic efficacy of GExos, wounds and ulcers were induced in B6.BKS(D)‐Leprdb/J (db/db) mice. The wounds were then treated with encapsulated GExos in sterile dressings that were replaced daily. The treatment was continued until complete re‐epithelialization of the wounds was observed. Subsequent tissue retrieval and analysis revealed that GExos significantly promoted angiogenesis and improved the remodeling of diabetic wounds. Importantly, this treatment is highly biocompatible and safe. In conclusion, GExos exhibit promising phyto‐nanotherapeutic properties, offering a novel approach for stimulating the healing of diabetes‐induced ulcers.^200^, ^201^ Their potential in clinical settings is significant as they may help reverse hyperglycemic dysregulation, promote wound re‐epithelialization, and stimulate vascular regeneration.

### 6.4 Colitis

IBD, which includes Crohn's disease and ulcerative colitis, presents with clinical signs such as elevated intraepithelial lymphocytes, surface inflammatory infiltrates, and epithelial damage.^202^, ^203^ The key factors in the pathogenesis of IBD include aberrant inflammatory responses, diminished levels of reactive oxygen species, and disrupted gut microbiota. These factors contribute to the initiation and progression of the intestinal inflammation. Medicinal plants, known for their natural ability to modulate inflammatory factors, are increasingly recognized for their potential in the clinical management of IBD.^204^, ^205^ The therapeutic effects of these plants are believed to be partially mediated by plant‐derived EVs. EVs can modulate intercellular communication within the complex inflammatory and microbial milieu of the gut. This modulation can strengthen the intestinal barrier and promote tissue repair, thereby alleviating IBD symptoms. The influence of plant‐derived EVs on intestinal homeostasis is a rich area of research with the potential to uncover novel therapeutic strategies for the clinical treatment of IBD. The exploration of these natural nanocarriers could pave the way for innovative and targeted interventions that harness the synergistic effects of plant bioactive compounds and EV‐mediated cellular communication.^206^

Yang et al.^207^ successfully isolated and purified ginseng‐derived nanoparticles (GDNPs) from ginseng roots using differential centrifugation. The yield of GDNPs was approximately 300 mg/kg of ginseng root,^208^, ^209^ indicating a substantial yield. These nanoparticles have been shown to scavenge reactive oxygen species from the intestinal epithelium and downregulate levels of M1‐type macrophages. The underlying mechanism involves the regulation of the TLR4/MAPK pathway, thereby upregulating the expression of anti‐inflammatory factors. Collectively, GDNPs reduced inflammatory mediators and regulated the intestinal microenvironment. In a separate study, Liu et al.^210^ isolated and characterized a specific population of turmeric‐derived nanoparticles (TDNPs 2). Using female FVB/NJ mice, a colitis model was established to explore the therapeutic mechanisms of TDNPs 2 in targeting IBD. In vivo studies have shown that the delivery of TDNPs 2 effectively targets inflammatory cytokines, thereby improving IBD in mice. In summary, these collective findings suggest that plant‐derived nanoparticles, such as GDNPs and TDNPs 2, offer a promising and safe therapeutic strategy for the clinical management of IBD.^211^, ^212^ The ability of natural nanocarriers to modulate inflammatory responses and improve the intestinal environment highlights the need for further studies in this area.

## 7 CHALLENGES AND PERSPECTIVES

Plant‐derived EVs are gaining attention because of their natural properties, safety, and efficacy. These vesicles are rich in biologically active components and exhibit various pharmacological effects.^213^, ^214^ They effectively target damaged cells and promote regeneration and repair.^215^ Additionally, plant‐derived EVs pass from the gut into the human bloodstream to treat clinical diseases by modulating intestinal flora and facilitating cellular exchange. As natural nanocarriers, EVs can be modified to carry drugs, enhancing their ability to target specific cells and tissues. Therefore, plant‐derived EVs have promising therapeutic potential for clinical and biomedical advancements.

However, despite their potential, several challenges remain. First, production scalability poses a significant obstacle to the implementation of plant‐sourced EVs into practice. The preparation and purification processes can be complex, necessitating advanced techniques such as ultrafiltration, PEG‐based precipitation, and size‐exclusion chromatography to improve purity and yield. These methods can be used to isolate specific EV subpopulations for various clinical applications. Therefore, new purification strategies must be developed to ensure scalability while maintaining quality. Establishing these techniques will provide a solid foundation for the future clinical treatment of diverse diseases.^216^, ^217^

Second, regulatory barriers pose significant challenges for the development of plant‐derived EVs. As this field emerges, many fundamental studies and clinical trial data remain unavailable, leading to uncertainty that makes regulators cautious about their approval processes.^218^ Currently, standardized guidelines are lacking for the extraction, purification, characterization, and quality control of plant‐derived EVs, which are crucial for their stable and efficient production.^219^ This inconsistency hampers data comparability between research institutions and complicates regulatory oversight. Therefore, it is vital to establish clear purification guidelines and regulatory standards to guarantee low toxicity of plant‐derived EV for disease treatment.^220^

Third, although plant‐derived EVs are generally regarded as nonimmunogenic, their potential immunogenicity deserves consideration, especially with repeated administration to patients with weakened immune systems. Over time, EVs accumulate in the body, possibly triggering immune responses. To mitigate this risk, optimizing dosing regimens, such as adjusting dosage and intervals, is essential for minimizing EV accumulation. Developing individualized treatment protocols is crucial, particularly for patients, such as organ transplant recipients, who may be more susceptible to external stimuli, developing individualized treatment protocols is crucial.^221^, ^222^ Additionally, stability during storage and transportation is vital for the practical application of plant‐derived EVs; therefore, selecting suitable cryoprotectants for different plants is important for optimizing their efficacy.^223^, ^224^

Finally, the development of targeted delivery systems is crucial for future research. Precise delivery not only enhances therapeutic efficacy, but also reduces off‐target effects, improving both safety and efficiency. Current strategies for optimizing EV delivery include surface modifications and genetic engineering techniques.^225^ Targeted delivery can be achieved by attaching specific ligands such as antibodies, peptides, or small molecules to the surface of EVs. Furthermore, the genetic engineering of plant cells can produce EVs that naturally carry targeting molecules.^226^ With continuous progress in nanotechnology and biotechnology, enhancing the precision of plant‐derived EV delivery to specific cells or tissues will become increasingly feasible, thereby boosting therapeutic efficacy and minimizing off‐target effects.^227^, ^228^ Therefore, there is an urgent need to identify novel separation strategies and safe drug delivery methods. Thus, an efficient and economical delivery system using EVs will pave the way for future clinical treatments.^210^

## 8 CONCLUSION

In summary, plant‐derived EVs have a huge potential for change in the field of medicine. It is especially valuable in clinical applications because of its lipid bilayer structure, which allows it to easily penetrate target cell membranes. They treat diseases through various mechanisms, including influencing intercellular communication, improving intestinal and tumor microenvironments, modulating immune‐inflammatory responses, and inhibiting apoptosis. Understanding the mechanisms of plant EVs in disease treatment will provide a theoretical foundation for their clinical applications. Therefore, it is crucial to explore new isolation strategies and safe administration methods in order to provide efficient and cost‐effective solutions for future therapies.

## AUTHOR CONTRIBUTIONS

Jinglin Wang, Haozhen Ren, and Peng Li conceptualized the idea and structured the article. Yawen Zhu, Junqi Zhao, and Haoran Ding conducted literature reviews and composed the manuscript. Gaolin Wen, Mengdi Qiu, Lingling Xue, and Dongxue Ge contributed to the writing process. Jinglin Wang oversaw the manuscript. All authors have read and approved the final manuscript.

## CONFLICT OF INTEREST STATEMENT

The authors declare no conflict of interest.

## ETHICS STATEMENT

Not applicable.

## Data Availability

The data that support the findings of this study are available on request from the corresponding author.
